# Urogenital tract and rectal microbiota composition and its influence on reproductive outcomes in infertile patients

**DOI:** 10.3389/fmicb.2023.1051437

**Published:** 2023-02-10

**Authors:** Yong-Hong Dong, Zhong Fu, Ning-Nan Zhang, Jing-Yi Shao, Jie Shen, En Yang, Shi-Yi Sun, Zhi-Min Zhao, An Xiao, Chen-Jian Liu, Xiao-Ran Li

**Affiliations:** ^1^Faculty of Life Science and Technology, Kunming University of Science and Technology, Kunming, Yunnan, China; ^2^Urology Department, The First People's Hospital of Yunnan Province, Kunming, Yunnan, China; ^3^Urology Department, The Affiliated Hospital of Kunming University of Science and Technology, Kunming, Yunnan, China; ^4^Department of Reproductive Medicine, The First People's Hospital of Yunnan Province, Kunming, Yunnan, China; ^5^Reproductive Medical Center of Yunnan Province, The Affiliated Hospital of Kunming University of Science and Technology, Kunming, Yunnan, China; ^6^Department of Infectious Diseases and Hepatic Disease, The First People’s Hospital of Yunnan Province, Kunming, Yunnan, China; ^7^Department of Infectious Diseases and Hepatic Disease, The Affiliated Hospital of Kunming University of Science and Technology, Kunming, Yunnan, China

**Keywords:** follicular fluid, infertile, microbiota, rectum, urogenital tract

## Abstract

**Introduction:**

Microbiota in the human body are closely related to human diseases. Female urogenital tract and rectal microbes have been considered as important factors affecting female pregnancy, but the mechanism is unknown.

**Methods:**

Cervical, vaginal, urethral, and rectal swabs were collected from 22 infertile patients and 10 controls, and follicular fluid was extracted from 22 infertile patients. The microbial composition of different sampling sites of infertile patients was examined. By comparing the microbial composition difference between infertile patients and controls and combining bioinformatics methods to analyze the potential impact of the female urogenital tract (cervical, vaginal and urethral) and rectal microbial diversity on female infertility and pregnancy outcomes.

**Results:**

*Lactobacillus* predominated in the female urogenital tract, but its abundance decreased in infertile patients, whereas the abundance of *Gardnerella* and *Atopobium* increased. The microbial changes in the urethra had the same trend as that in the vagina. Compared with healthy controls, the cervical and rectal microbial diversity of infertile patients were significantly increased and decreased, respectively. There might be interactions between microbes in different parts of female. *Geobacillus thermogeniticans* was enriched in the urogenital tract and rectum of infertile patients, and has a good predictive effect on infertility. Compared with infertile patients, *L. johnsonii* was enriched in the vagina, urethra, and intestine of the control group. *L. acidophilus* in follicular fluid might be associated with Non-pregnancy.

**Conclusion:**

This study found that the microbial composition of infertile patients was changed compared with that of healthy people. The translocation of Lactobacillus between the rectum and urogenital tract might play a protective barrier role. The changes of *Lactobacillus* and *Geobacillus* might be related to female infertility or pregnancy outcome. The study provided a theoretical basis for the future treatment of female infertility from the perspective of microorganisms by detecting the microbial changes associated with female infertility.

## Introduction

There are abundant microorganisms (known as the microbiota) that live inside the human body and on the skin surface, which far outnumbers the body’s somatic cells (Turnbaugh et al., 2007). Human microbes can be divided into three categories-beneficial, pathogenic and opportunistic and they play a vital role in maintaining human health (Panthee et al., 2022). The change of microbial diversity in the human body is related to diseases in various parts of the human body, including the skin, gastrointestinal tract, urinary tract, reproductive tract, and other disease-prone parts (Binek, 2012). Therefore, discovering the characteristics of microbiome differences between health and disease will facilitate the diagnosis of related diseases and may provide new ways to prevent disease onset or improve prognosis.

The intestinal microbiota was thought to change host immunity by regulating multiple immune pathways, thus affecting the treatment results of various diseases (Gopalakrishnan et al., 2018; McQuade et al., 2019). Studies had found that changes in intestinal microbial diversity can also have an impact on gynecological diseases (Ata et al., 2019; Quaranta et al., 2019; Yurtdas and Akdevelioglu, 2020). In recent years, in addition to the gastrointestinal niche, more and more attention has been paid to the study of the human microbial state, the microbiota related to women’s reproductive health and the health of their offspring (Younes et al., 2018). In the last decade, the Human Microbiome Project has enabled the study of the structure and composition of the microbiome at different body sites, revealing that the female reproductive tract microbiota accounts for approximately 9% of the total bacterial load in humans (Moreno and Simon, 2019). It is well-established that the vagina is colonized by bacteria that serve important roles in homeostasis (Green et al., 2015). The study divided the vaginal microbiota of healthy women into five groups, four of which were mainly *Lactobacillus* spp. indicating that a potential key ecological function, the production of lactic acid, seems to be conserved in all communities (Ravel et al., 2011). Imbalances in the proportion of bacteria may lead to a predisposition to infection or reproductive complications (Green et al., 2015). The majority of studies on the female reproductive tract microbiome are focused on the vagina because of the ease of sampling; yet, several studies have demonstrated the existence of bacterial colonization beyond the vagina, showing that the upper reproductive tract is not sterile (Moreno and Simon, 2019). Chen et al. found distinct microbial communities in cervical canal, uterus, fallopian tubes, and peritoneal fluid, differing from that of the vagina and the results reflected a microbiota continuum along the female reproductive tract, indicative of a non-sterile environment (Chen et al., 2017). Due to the characteristics of the female reproductive structure, the anatomical location of the genital tract, urinary tract, and intestinal tract is often conducive to microbial co-colonization. Brown et al. found substantial agreement in the composition of the microbiota between reproductive-aged women’s urine samples and paired mid-vaginal swabs (Brown et al., 2021). Komesu et al. also demonstrated a significant association between the vaginal and urinary microbiomes, with *Lactobacillus* predominance in both urine and vagina.

Infertility, defined as 1 year of attempted conception without success, is one of the most prevalent chronic health disorders involving young adults (Smith et al., 2003). The major causes of female infertility are anovulation, fallopian tube disease, pelvic adhesions, endometriosis, and unexplained infertility (Barbieri, 2019). Due to the complex causes of infertility, its long treatment cycle and high cost of treatment often have a great impact on people’s lives. *In vitro* fertilization is the most common treatment for infertility, but its low success rate often brings a double blow of economic and mental pressure to patients (Margan et al., 2022). At present, the main methods to solve the problem of female infertility are to identify the complex influencing factors of infertility and improve the success rate of *in vitro* fertilization.

Sometimes people often face infertility due to sudden exposure to exogenous microbial pathogens either of gram-positive or gram-negative origin. The infectious sites include different organs of the genital tract irrespective of gender (Javed et al., 2022). The previous study have found that improving dysregulated intestinal flora was beneficial to increase the success rate of pregnancy in females with infertility (Komiya et al., 2020). More *Prevotella*, *Streptococcus*, and *Fusobacterium* were detected in the intestines of infertile patients (Lan et al., 2022). With the deepening of the research on female reproductive tract microbe and female health, more and more studies have linked female infertility with reproductive tract microbe. Bacterial infections of the female reproductive tract, including vaginitis, cervicitis, and endometritis, have been recognized as a threat to fertility in many species (Heil et al., 2019). The microbiome of the female reproductive tract is involved in the physiology and patho-physiology of reproduction, affecting a broad range of processes from gametogenesis to fertilization, embryo migration, and/or implantation (Garcia-Grau et al., 2019). In a study of the vaginal microbes of women who had received embryo transfers, women with a low percentage of *Lactobacillus* in their vaginal sample were less likely to have a successful embryo implantation (Koedooder et al., 2019). Clinical pregnancy in fresh *in vitro* fertilization and embryo transfer cycles might be affected by cervical microbial composition (Hao et al., 2021). Pelzer et al. thought microorganisms colonizing follicular fluid and the ensuing cytokine response could be a further as yet unrecognized cause and/or predictor of adverse assisted reproduction technology outcomes and infertility. Therefore, a better understanding of the changes in the composition of reproductive tract microorganisms in infertile women will help us identify potential microbial markers related to female pregnancy and reproductive outcomes, and provide a theoretical basis for the prevention and even treatment of female infertility from the perspective of microorganisms. This study characterized the urogenital tract and rectal microorganisms of infertile women, compared them with healthy women, and outline the change features of urogenital tract and rectal microorganisms of infertile women and the relationship with pregnancy outcome, which has certain guiding significance for the evaluation and treatment of female infertility.

## Materials and methods

### Patient recruitment

This study recruited 22 infertile female patients undergoing IVF treatment and 10 healthy control (Asymptomatic childbearing aged women who receive for a routine well woman visit or preconceptional counselling) at the Department of Reproductive Medicine of The First People’s Hospital of Yunnan Province, China, in 2021. To reduce the impact of climate and hospital environment in different periods, all samples were collected within 1 month. Inclusion criteria were as follows: 20–40 years of age and all subjects (infertile group and healthy control group) did not receive antibiotics or any antimicrobial treatment within 3 months and did not use hormonal contraception and no sexually transmitted infection and urinary tract infection. Exclusion criteria were as follows: male factor; autoimmune diseases; endocrine diseases. Having clinical signs and symptoms suggestive of infertile, i.e., dysmenorrhea, dyspareunia, Menstrual disorder and/or amenorrhea precluded recruitment to the control group. All healthy controls had normal pregnancy history. Participants understood the nature of the study, explicitly agreed to provide their personal information, and provided written understanding.

### Sample collection

To avoid potential effects of sedatives or anesthetics used for oocyte retrieval in infertile patients, all swab samples were collected within 3 days before oocyte retrieval. In the operating room, swabs were taken from the rectal, vaginal, and urethra of the participants. The participants was token the bladder lithotomy position, the sterile vaginal speculum was inserted and the cervical cell shedding fluid was collected by Cytobrush by rotating 360° in the cervix. “All infertile patients began to receive ovarian hyperstimulation treatment on the 5th day of the menstrual cycle. Follicular development was monitored by B-ultrasound. When the diameter of follicles reached 18 mm, drugs such as hCG were given to make multiple follicles discharge and oocyte retrieval were undertaken in the proliferative phase of the menstrual cycle.” Follicular fluid was collected using a sterile negative pressure needle. During this process, sampling was performed by a professional medical workers to ensure that the Cytobrush and negative pressure needle did not contact the cervix or vaginal wall of the participant, and most likely to avoid vaginal or cervical contamination. All of the specimens were collected in sterile tubes, then flash-frozen with liquid nitrogen, stored at −80°C prior to DNA extraction.

### DNA preparation, PCR amplification, and sequencing

Sample processing and DNA extraction works were performed meticulously in a strictly controlled and sterile environment. A strict negative control was set up in the experiment, and the laboratory sterile water was selected as the control to participate in the subsequent experiment to detect whether there was any pollution problem in the swabs, centrifuge tube (both swab and centrifuge tube used in the control experiment were exposed to the sampling environment), reagents, consumables used for DNA extraction and the operation process. All 4 ml of the cervical cell shedding fluid and follicular fluid sample was centrifuged for 10 min at 12,000 r/min, and the pellet was used for vagina, bowel and urethra DNA extraction. Microbial DNA was extracted all of samples using the QIAamp Pro Prowerfecal DNA Kit (Qiagen, Hilden, Germany) according to the manufacturer’s protocol. DNA yield was assessed using Nanodrop 2000 (Thermo Fisher, United States). Primers 515F (Reysenbach et al., 1992) and 909R (Brunk and Eis, 1998) were used for PCR amplification. All primers used containing Illumina adapters sequences and dual-index barcodes to distinguish each sample. For each PCR reaction, 15 ng DNA was added as template. The PCR reaction conditions were as follows: predenaturation at 95°C for 15 s, followed by 30 cycles of 95°C for 3 min, annealing at 51°C for 30 s, extension at 72°C for 30 s, then a final extension step at 72°C for 5 min. During the DNA extraction and PCR batch for each sample, the same reagents and consumables were used, and the PCR amplification procedure was the same for all DNA, including negative controls. No PCR bands were detected in all negative control samples. The amplicon were purified with UltraClean PCR Cleanup Kit (MOBIO, United States), and the equivalent amount of PCR products were sequenced in the Illumina Miseq^™^ system (Illumina, United States).

### Sequence analysis and statistics

All sequences were demultiplexed using the barcodes of each sample. The standardization of the sequences was processed using Mothur software. SILVA (V138) database was downloaded from Mothur website for sequence alignment and classification. Chimerism checking was performed after sequence alignment. OTUs were clustered according to the minimum homology threshold of 97%.

Measurements of α diversity (within sample diversity) such as observed, Shannon index, Simpson index, ACE index, Chao1 index and OTU numbers were calculated at OTU level using the Mothur. GraphPad was used for statistical analysis and *p*-value was set to be 0.05. The analysis of β-diversity (diversity between samples) was calculated by the Bray Curtis dissimilarity measure with Mothur and expressed by principal component analysis (PCA). Linear Discriminant Analysis (LDA) Effect Size (Eckburg et al., 2005) was used to identify the differential OTUs responsible for the discrimination between the differential groups. The “psych” package in R was used to calculated spearman’s rank correlation and *p* value, and the “pheatmap” package was used for plot visualization. The co-expression network was constructed with Gephi software. The receiver operating characteristic curve (ROC) was constructed with the “pROC” package. Pearson correlation coefficient was used to describe different OTUs, environmental factors and the correlation between OTU and environmental factors. Mantel correlations between microbial compositions and environmental data based on UniFrac distance (9,999 permutations) were calculated using the pure R software package.

### Nucleotide sequence availability

The raw data of the article entitled “Urogenital tract and rectal microbiota composition and its influence on reproductive outcomes in infertile patients” had been uploaded to The National Omics Data Encyclopedia (NODE) at https://www.biosino.org/node/, accession number: OEP003637.

## Results

### Distinct microbiota abundance and composition within infertile and health female

The cervical, vaginal, rectal, and urethral samples of 32 female participants (22 infertile and 10 controls) were included in the study, and the follicular fluid of infertile patients was collected (the follicular fluid of the control group was refused to be collected). Among them, the follicular fluid samples from 2 infertile patients and the cervical samples from 9 infertile patients failed to be sequenced due to the low DNA content. Table 1 summarizes the baseline characteristics of the subjects. In the case of similar height, the BMI of the control group was significantly higher than that of the infertile patients, and the infertile patients had a lighter weight.

**Table 1:** The baseline characteristics of the subjects.

	Infertility	Control	*P*
Number	22	10	–
Mean age (year)	31.4 ± 4.7	33.8 ± 4.5	0.20
Height	160.0 ± 5.9	158.9 ± 4.8	0.59
Weight	55.5 ± 7.0	61.6 ± 7.7	0.08
BMI^1^	22.1 ± 2.7	24.4 ± 3.0	0.04*
STIs^2^			–
Yes	0	0	
No	22	10	
BV^3^	3	0	–
Abortion history			0.05
Yes	9	0	
No	13	10	

* indicates the difference between infertility and control groups, *p* < 0.05.

1. Body Mass Index, 2. Sexually transmitted infections, 3. Bacterial vaginosis.

**Figure 1:**
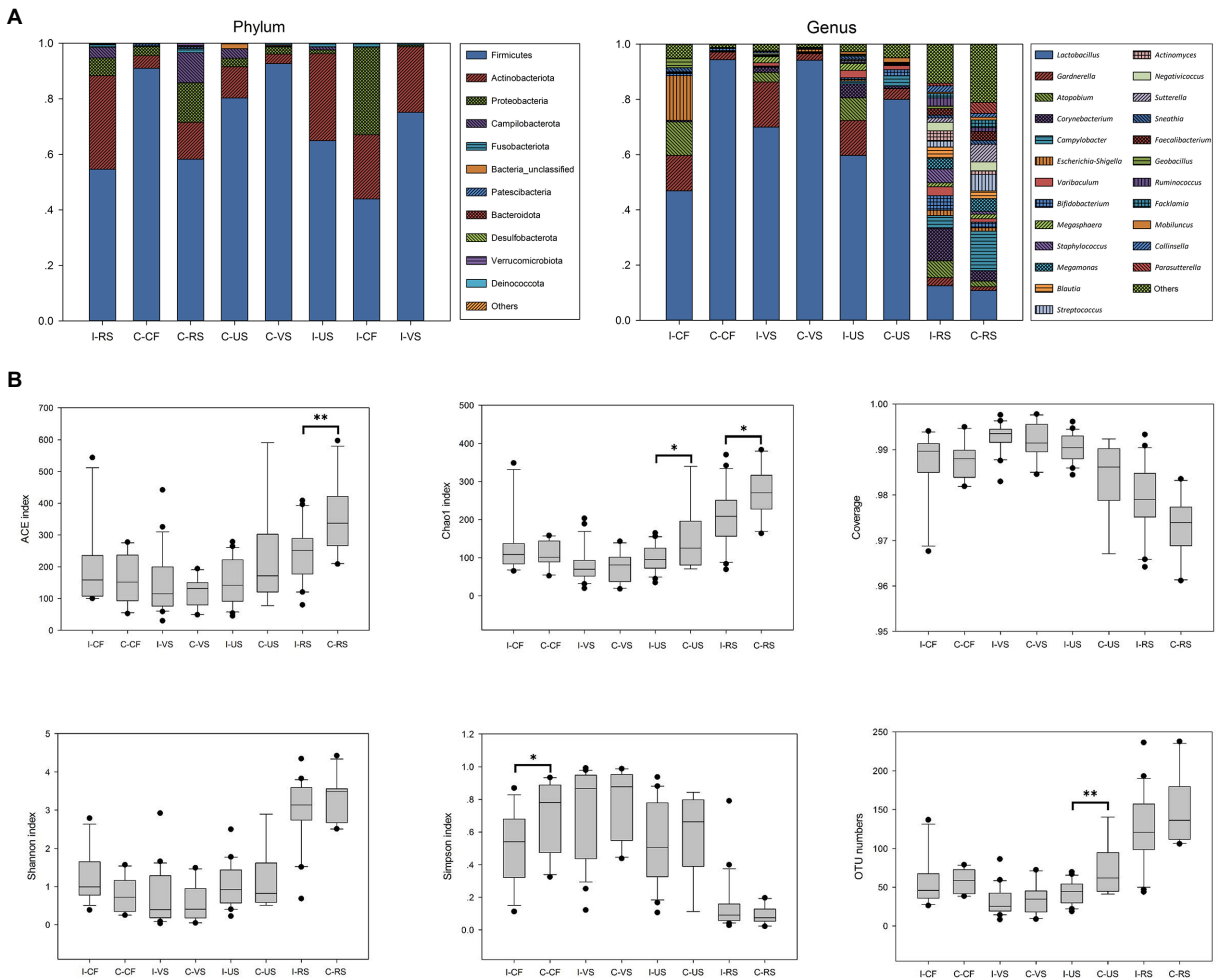
Microbial composition and diversity analysis of urogenital tract and rectal. **(A)** The composition ratio of each microorganism at phylum and genus level, **(B)** Alpha diversity between different sampling sites based on the OTU numbers, Shannon, Simpson, Chao1, ACE indices and Coverage. *t*-test, *p* values represented as *p < 0.05, **p < 0.01, ***p < 0.001. I-*CF*, Infertile patients – Cervical fluid; C-*CF*, Controls – Cervical fluid; I-*VS*, Infertile patients – Vaginal swabs; C-*VS*, Controls – Vaginal swabs; I-US, Infertile patients – Urethra swabs; C-US, Controls – Urethra swabs; I-RS, Infertile patients – Rectal swabs; C-RS, Controls – Rectal swabs.

Here, 16S rRNA gene sequencing was used to compare the microbial composition of patients with infertile and controls. The relative abundances of different sampling sites at Phylum and Genus levels in both groups were described in Figure 1A. Firmicutes were the most abundant phylum of all samples and Actinobacteriota had been detected more frequently in infertile patients. At genus level, Compared with the healthy control group, the abundance of *Lactobacillus* in vagina, cervix, and urethra of infertile patients decreased, while the abundance of *Gardnerella* and *Atopobium* increased.

Through the comparison of α-diversity index (OTU numbers and ACE, Chao1, Shannon, and Simpson indices), it was found that there were differences in microbial diversity index at different sampling sites (Figure 1B), and there were also differences between infertile patients and controls. Generally, rectal α diversity index was the highest, and vaginal diversity was lower than other samples. The diversity of cervix microbes in infertile patients was significantly higher than that in controls, while the abundance of microbes in rectal and urethra was significantly lower than that in controls (*p* < 0.05).

### OTU-based analysis of different sampling sites in infertile and controls

**Figure 2:**
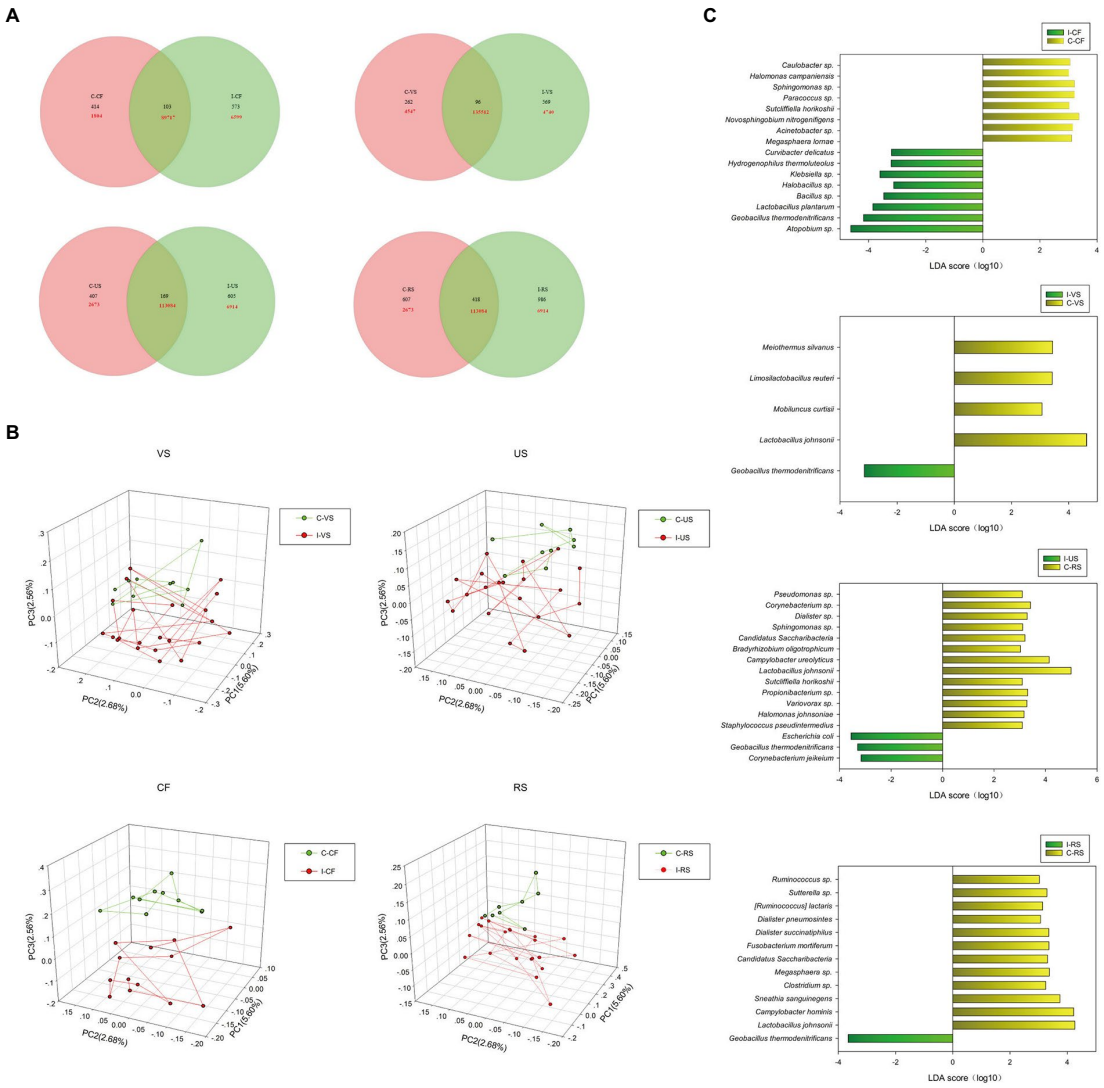
Differences in microbial diversity of cervix, vagina, urethra, rectal between infertile patients and controls group. **(A)** Venn diagram shows the OTUs and sequences shared by different groups at different sampling sites. **(B)** PCA plot based on the relative taxon abundance in cervix, vagina, urethra and rectal samples from infertility and control groups. **(C)** LEfSe analysis detected signature microorganisms at different sampling sites, LDA > 2, yellow shows the positive LDA score indicating enrichment in infertility patient samples; gray shows the negative LDA score indicates the taxa enriched in the control sample.

Based on OTU data, the differences in microbial composition in four sampling sites of infertility patients in this study were analyzed. First, Venn diagram analysis showed that infertile patients and controls shared the least number of sequences in vagina (91.4%) and the least number of OTU in cervix (Figure 2A). As shown in Figure 2B, the Principal components analysis (PCA) found that the infertile patients could be differentiated from the controls by their cervix microbiota (*p* < 0.05). The distribution of rectal and urethral samples in infertile patients was also different from that in the control group.

**Figure 3:**
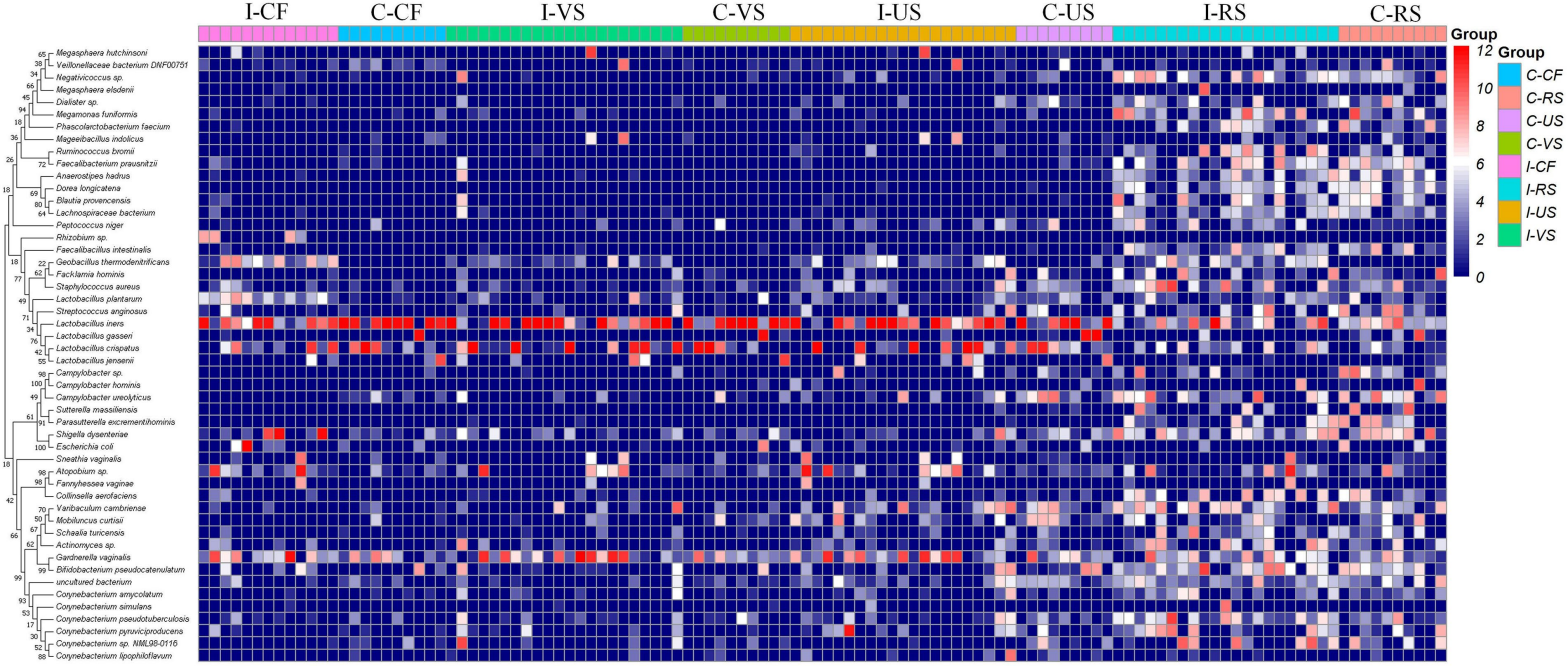
Combined diagram of heatmap and phylogenetic tree were constructed for the most abundant 50 OTUs, cervical fluid, vagina, urethra and rectal swabs of different groups were, respectively, show with different colors.

Detailed information and specific distributions for the top 50 OTUs in all samples are shown in Figure 3. There were significant differences in the distribution of major OTUs between urogenital tract and rectum. *L. iners* was detected in a large number of urogenital tract samples. *L. crispatus* was mainly distributed in the control group, while *Gardnerella vaginalis, Atopobium* sp., and *Geobacillus thermodenitrificans* were more abundant in the infertile patients.

Considering that there were statistical differences in α diversity and β diversity between infertile patients and controls, a LEfSe analysis was performed to identify the potential bacterial candidates as biomarkers associated with infertility disease. As shown in Figure 2C, A total of 17 different species were identified in the cervix, of which *Atopium* sp., *Geobacillus thermodenitrificans*, *L. plantarum*, *Bacillus* sp., *Halobacillus* sp., *Klebsiella* sp., *Hydrogenophilus thermoluteolus*, *Curvibacterium delicatus* were enriched in infertile patients, *Megasphaera lornae*, *Acinetobacter* sp., *Novosphingobium nitrogenigenicals*, *Sutcliffiella horikoshii*, *Paracoccus* sp., *Sphingomonas* sp., *Halomonas campaniensis*, *Caulobacter* sp., *Sphingomonas* sp. in controls. Five different species were identified in the vagina, among which *Lactobacillus Johnsonii*, *Mobiluncus Curtisii*, *Limosilactobacillus Reuteri*, *Meiothermus Silvanus* were enriched in the control group, *Geobacillus thermodenitrificans* were enriched in the infertility group. Sixteen different species were identified in the urethra, of which *Corynebacterium jeikeium*, *Geobacillus thermodenitrificans*, *Escherichia coli* were enriched in the infertility group, and Thirteen species such as *L. johnsonii*, *Campylobacter ureolyticus*, *Staphylococcus pseudotermedius* were enriched in the control group. Thirteen different species were identified in the rectum, of which *Geobacillus thermodenitrificans* were enriched in the infertility group, and 12 species such as *L. johnsonii*, *Campylobacter hominis*, and *Sneathia sanguinegens* were enriched in the control group.

### Correlation analysis of biomarkers at different sampling sites

**Figure 4:**
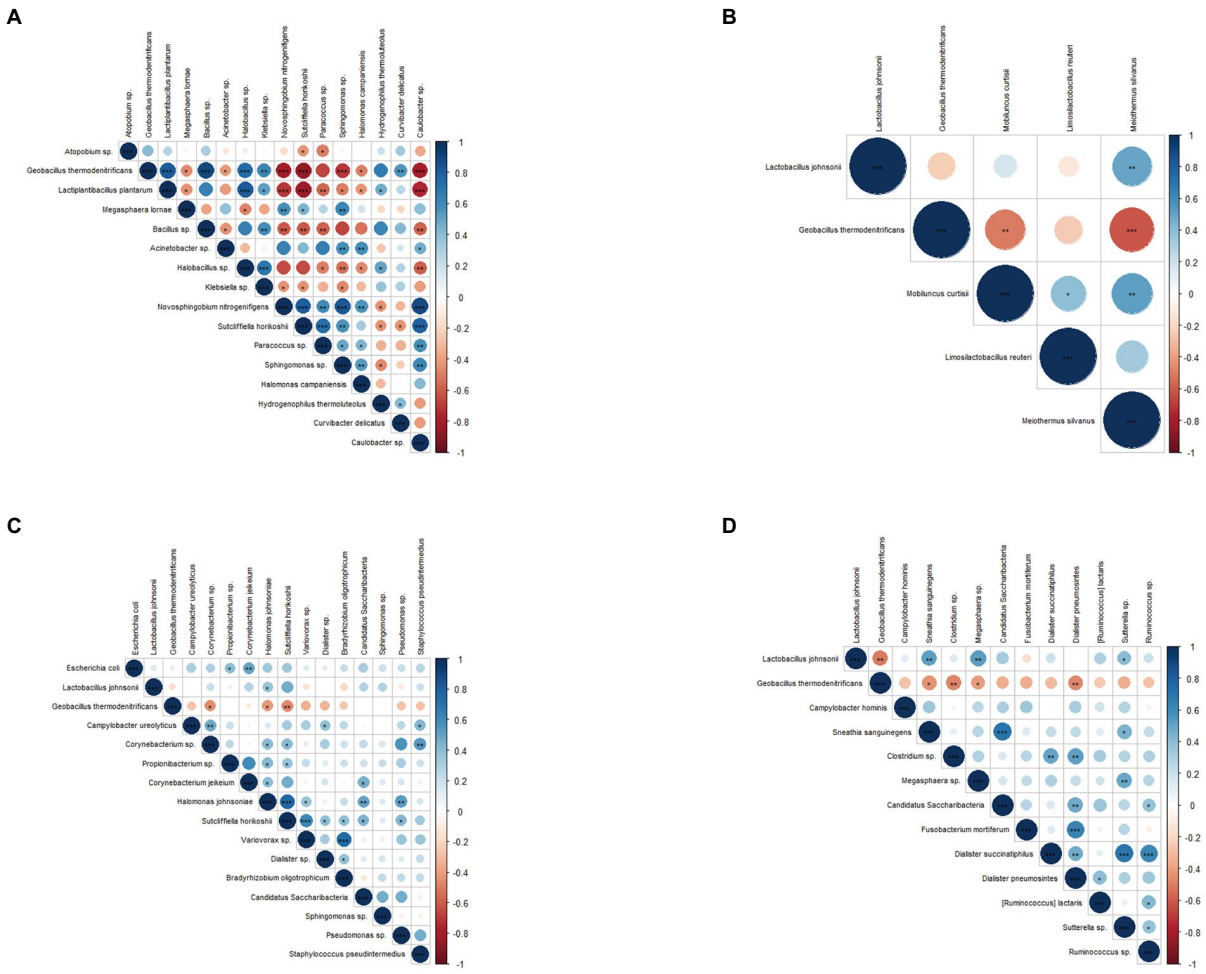
Correlation analysis of microbial markers between different sampling sites. **(A)** Spearman’s rank correlation analysis of microbial markers in the cervix, **(B)** in the vagina, **(C)** in the urethra, and **(D)** in the intestine. Blue indicates positive correlation, red indicates negative correlation, *p* values represented as *p < 0.05, **p < 0.01, ***p < 0.001.

Spearman correlation coefficient analysis of biomarkers detected at different sampling sites demonstrated that biomarkers within the same group were positively correlated, while those between groups were negatively correlated. Specifically, In the cervical, *Geobacillus thermodenitrificans*, *L. plantarum*, *Bacillus* sp., *Halobacillus* sp., *Klebsiella* sp., and *Curvibacter delicatus* in the infertile group were significantly positively correlated. *Caulobacter* sp., *Novosphingobium nitrogenifigens*, *Sutcliffiella horikoshii*, *Paracoccus* sp., and *Sphingomonas* sp. in the control group were significantly positively correlated (Figure 4A). In the vagina, *Geobacillus thermodenitrificans* was negatively correlated with *L. Johnsonii, Mobiluncus curtisii*, and *Meiothermus Silvanus* (Figure 4B). In the urethra and rectal, *Geobacillus thermodenitrificans* was negatively correlated with other species (Figures 4C,D). *L. Johnsonii* was negatively correlated with *Sneathia sanguinegens, Megasphaera* sp., and *Sutterella* sp. in the rectal (Figure 4D).

### Predictive ability of female infertility by urogenital tract and recta microbiome

**Figure 5:**
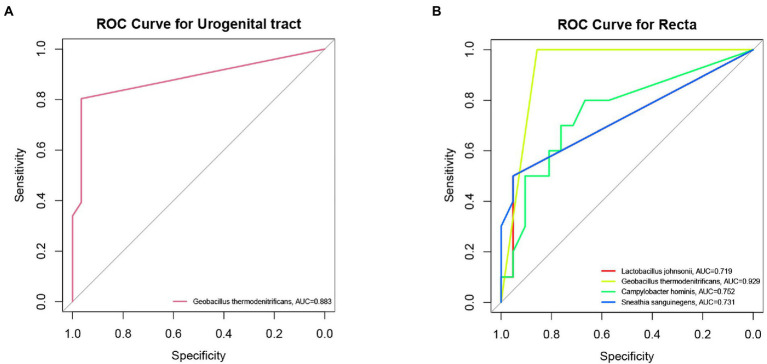
ROC analysis predicted major biomarkers that could distinguish infertile patients from controls. **(A)** Discrimination of *Geobacillus thermodenitrificans* in the urogenital tract (cervix, vagina, intestine), AUC = 0.883. **(B)** The discriminative effect of different rectal biomarkers (*Geobacillus thermodenitrificans*, AUC = 0.929; *Campylobacter hominis*, AUC = 0.752; *Sneathia sanguinegens*, AUC = 0.731; And *L. Johnsonii*, AUC = 0.719).

In order to explore the role of biomarkers in diagnosis, the samples of urogenital tract and rectal in infertility group and control group were analyzed by receiver operating characteristic. *Geobacillus thermodenitrificans* was observed in the urogenital tract had a discriminatory ability with AUC of 0.883 as shown in Figure 5A. Four microorganisms (*L. johnsonii*, *Geobacillus thermodenitrificans*, *Sneathia sanguinegens*, and *Campylobacter hominis*) were found as discriminators when ROC analysis was used to identify recta microorganisms in infertile patients and controls (AUC > 0.7), as shown in Figure 5B. Among the four species, *Geobacillus thermodenitrifican* showed the strongest diagnostic power (AUC = 0.929), followed by *Campylobacter hominis* (AUC = 0.752).

### Correlation analysis of microbiota between genitourinary tract and rectum

**Figure 6:**
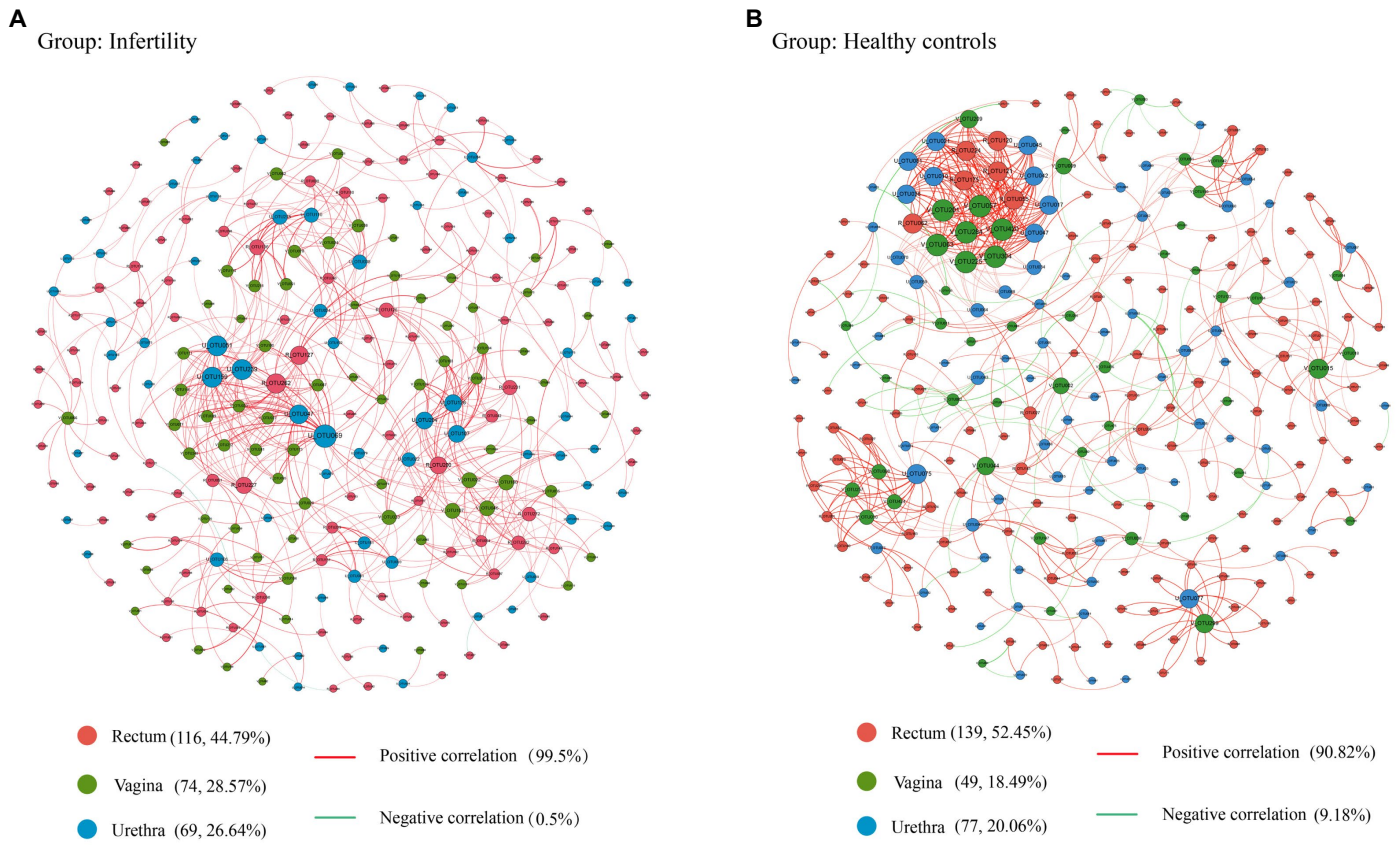
OTU correlation network diagram among three different parts of rectum, vagina, and urethra. **(A)** species interaction patterns among different parts in infertile patients, **(B)** species interaction patterns among different parts in healthy controls.

In this study, the correlation between OTUs was used to build a microbial community network to study the microbial interaction patterns between the rectum, urethra, and vagina of infertility patients and healthy control group. It can be seen from Figures 6A,B that there are different microbial interaction networks between infertile patients and healthy control groups. Rectal microbes have the largest number of nodes in the two groups’ networks. The majority of urogenital tract microbes in infertile patients were positively correlated. Further research found that OTU001 (*Lactobacillus iners*), OTU038 (*Mageeibacillus indolicus*), and OTU126 (*Corynebacterium sundsvallense*) all exist in the rectum, urethra of infertility patients, Vaginal sites, and each OTU had a positive correlation between these three different sites.

### The effects of urogenital tract microorganisms on the outcome of *in vitro* fertilization-embryo transfer

**Figure 7:**
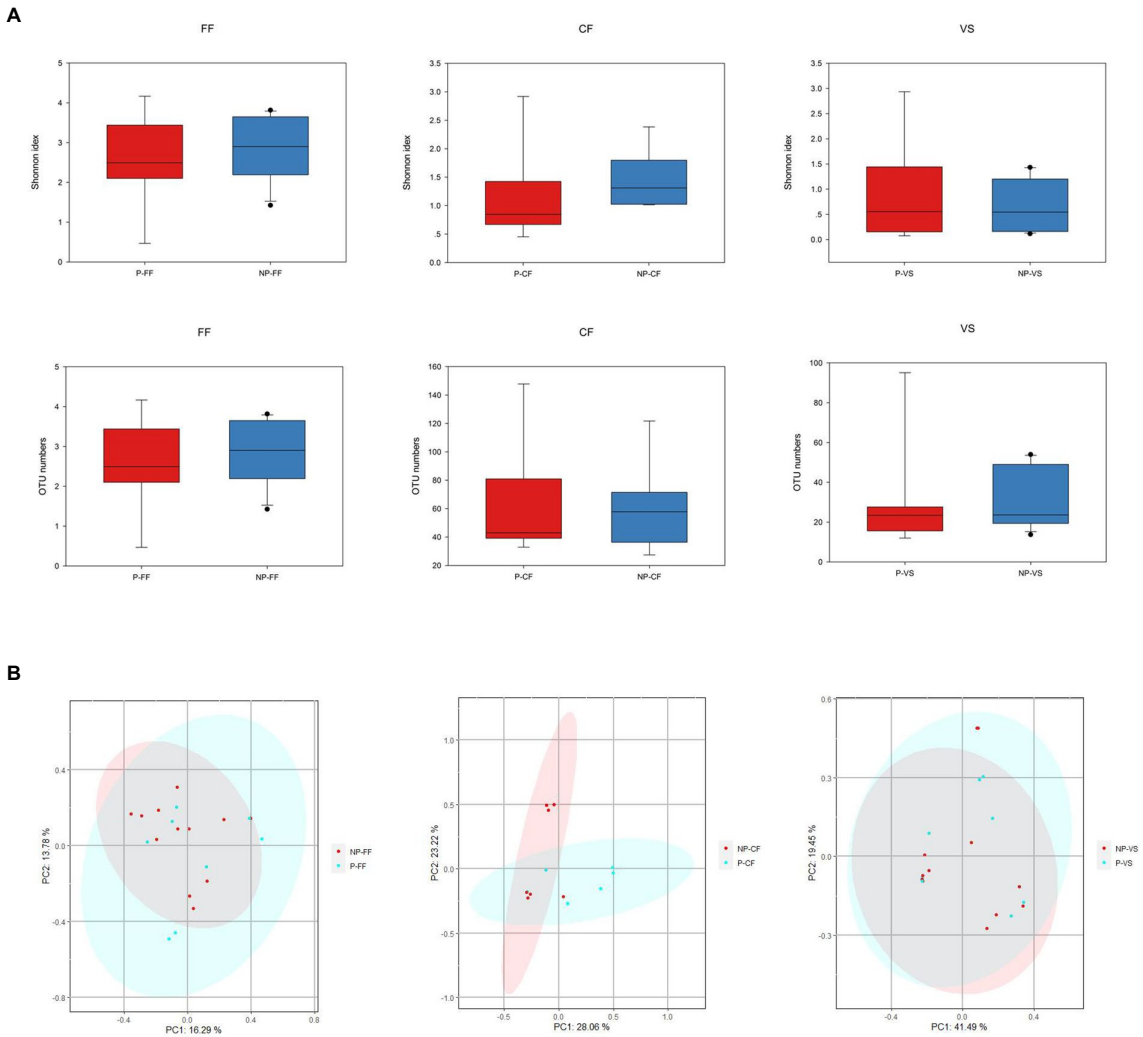
Microbial composition and differences of three reproductive tract sites of different pregnancy outcomes. **(A)** Microbial alpha diversity in Pregnancy and Non-pregnant groups, **(B)** At the OTU level, PCA analysis showed differences in microbial composition in different parts of the reproductive tract between the Pregnancy and Non-pregnant groups.

According to the pregnancy after embryo transfer, infertile women were further divided into two subgroups: Pregnant and Non-pregnant groups. Analyze the microbial differences in vagina, cervix, and follicular fluid. “Among the 22 infertile patients, 9 were positive and 13 were negative after embryo transfer, and the relevant clinical characteristics information was shown in Table 2. The age of the Pregnant group was significantly younger than that of Non-pregnant group, and the number of oocytes matures and quality embryos in Pregnant group were slightly higher than that in Non-pregnant group.” Alpha diversity analysis showed that the Shannon index of Non-pregnant group was higher than that of Pregnant group (Figure 7A). The principal component analysis (PCA) was used to compare the microbial communities in vaginal, cervical, and follicular fluid of Pregnant and Non-pregnant women. As shown in Figure 7B, there was no obvious separation of samples between the two groups and permannova tests showed no significant differences according to the condition status in all niches.

**Table 2:** The baseline characteristics of the infertile patients.

	Pregnant	Non-pregnant	*P*
Number	9	13	–
Mean age (year)	28.7 ± 3.1	33.3 ± 4.6	0.02*
Height	160.1 ± 6.1	160 ± 5.6	0.9
Weight	54 ± 7.1	58.3 ± 6.3	0.17
BMI^1^	21.1 ± 2.5	22.8 ± 2.6	0.14
Infertility diagnosis			0.27
Primary infertility	5	3	
Secondary infertility	4	10	
AMH^2^	3.65 ± 1.41	2.9 ± 1.6	0.34
TSH^3^	2.24 ± 1.2	2.5 ± 1.1	0.61
OMR^4^	0.78 ± 0.08	0.86 ± 0.1	0.07
MON^5^	11.7 ± 3.3	10.5 ± 6.3	0.64
Quality embryos	5.1 ± 1.4	4.2 ± 3.7	0.52
BV^6^	1	2	–
Abortion history			0.47
Yes	4	9	
No	5	4	

* indicates the difference between Infertility and Control groups, *p* < 0.05.

1. Body Mass Index, 2. Anti-Müllerian hormone, 3. Thyroid stimulating hormone, 4. Ovum maturation rate, 5. Mature ovum number, 6. Bacterial vaginosis.

**Figure 8:**
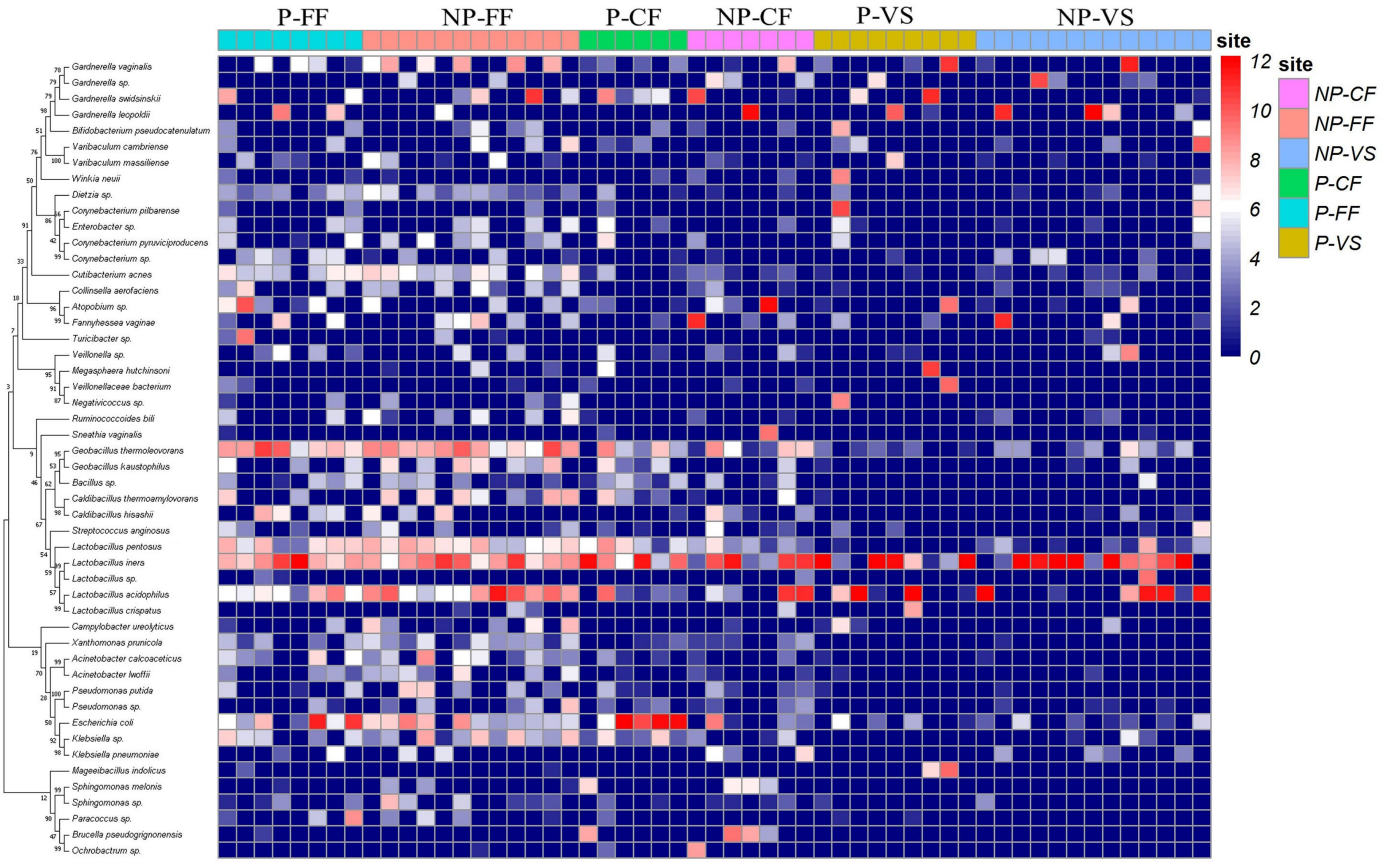
OTU heat map of the top 50 abundances in different samples. P-FF, Pregnancy follicular – Fluid; NP-FF, Non-pregnancy – Follicular fluid; P-*CF*, Pregnancy – Cervical fluid; NP-*CF*, Non-pregnancy – Cervical fluid; P-*VS*, Pregnancy – Vaginal swabs; NP-*VS*, Non-pregnancy – Vaginal swabs.

**Figure 9:**
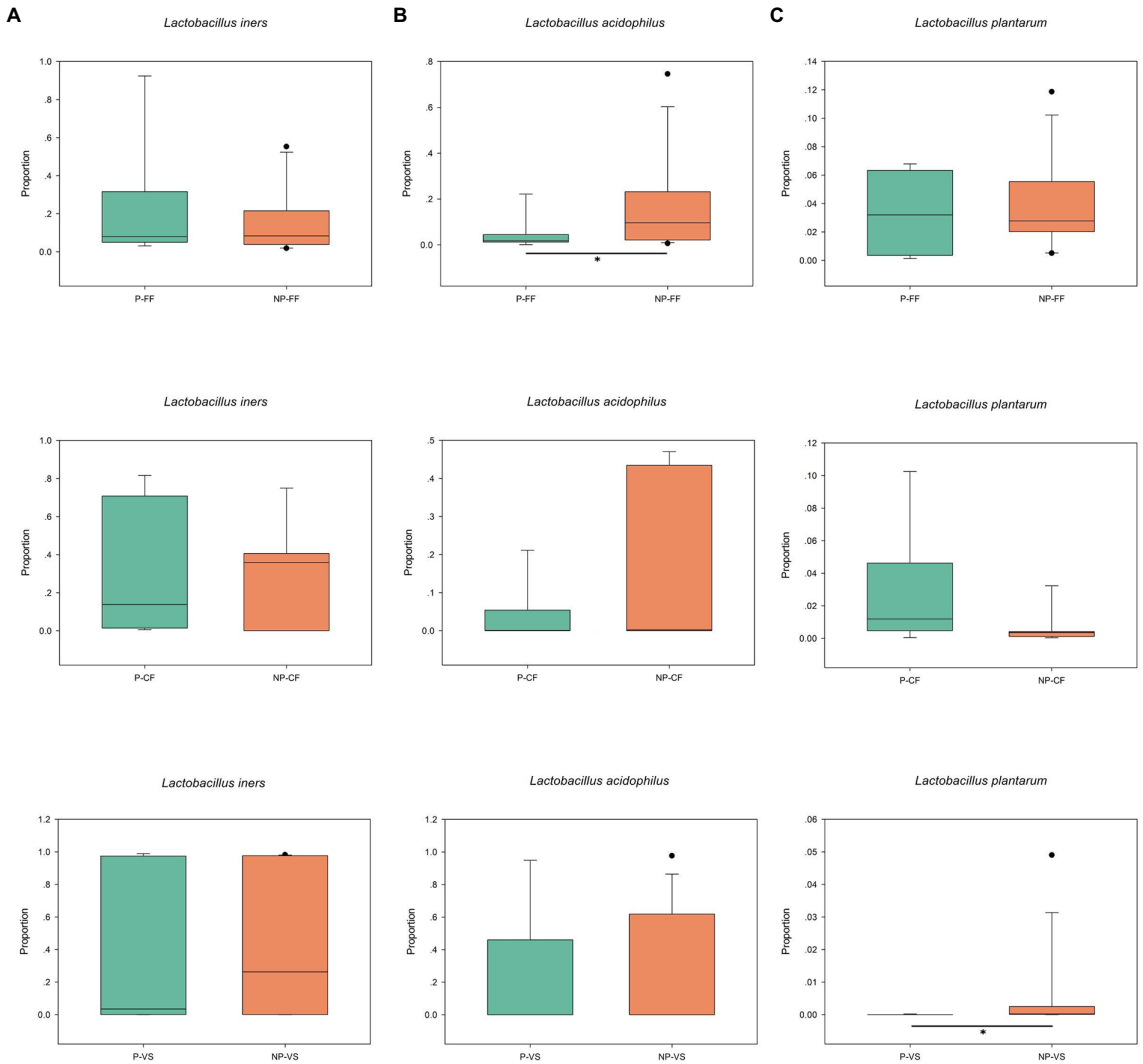
Relative abundance of different *Lactobacillus* species at three reproductive tract sites of different pregnancy outcomes. **(A)** Follicular fluid, **(B)** cervix, **(C)** vagina. The points on the top of the boxes are the outliers. Wilcoxon rank-sum test, *p* values represented as *p < 0.05, **p < 0.01.

**Figure 10:**
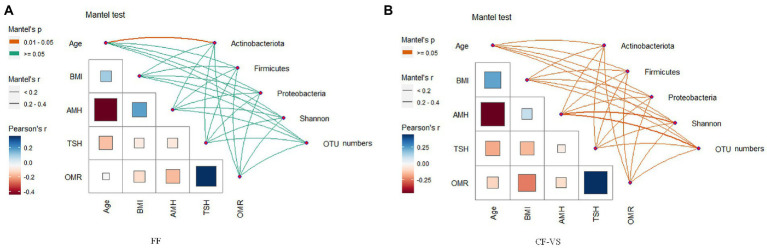
Paired comparison of environmental factors and microbial community. **(A)** Correlation analysis of phylum level microorganisms and diversity with environmental factors in follicular fluid, **(B)** Analysis on the correlation between environmental factors and microbial diversity at the level of cervix and vagina. The color of the line indicates the size of the significance p value of the Mantel test, and the thickness indicates the size of the correlation, FF, Follicular fluid; *CF*-*VS*, Cervical fluid – Vaginal swabs.

OTU analysis of the first 50 reproductive tract abundances (Figure 8) showed that the reproductive tract of infertile patients was dominated by *Lactobacillus*, among which *L. iner*s had the highest abundance, while *L. crispatus* was less detected. In vagina, the abundance of *L. iners* in Pregnancy group was lower than that in Non-pregnant group. In follicular fluid, In addition to *L. iners*, a certain abundance of *L. acidophilus, L. plantarum, Geobacillus*, and *Escherichia* was detected. At the species level, different *Lactobacillus* species were compared among the three sites (Figure 8). Specifically, *L. iners* was the most important lactic acid bacteria, and its proportion in Pregnant group was higher than that in Non-pregnant (Figure 9A). The proportion of *L. acidophilus* was higher in the Non-pregnant group. *L. acidophilus* was significantly enriched in follicular fluid of Non-pregnant group (Figure 9B; *p* < 0.05). *L. plantarum* was significantly enriched in the vaginas of Non-pregnant group (Figure 9C; *p* < 0.05). To identify the impact of environmental factors on microorganisms, analyzed the correlation between the dissimilarities of microbiological phylum level of urogenital tract, rectum, and those of environmental factors (Figure 10). In general, AMH (Anti-Müllerian hormone) was significantly negatively correlated with age, and TSH (Thyroid stimulating hormone) was positively correlated with OMR (Ovum maturation rate). Actinobacteriota in follicular fluid was significantly correlate1d with age (Figure 10A).

## Discussion

The female reproductive tract microbiome plays an essential role not only in health and dysbiosis but also potentially in successful fertilization and healthy pregnancies (Younes et al., 2018). This study assessed the microbiome community composition of the urogenital tract (vagina, cervix, urethra) and intestines of infertile patients and controls. At the same time, the microbiota differences in the female vagina, cervix, and follicular fluid under different pregnancy outcomes were discussed. This study showed the unique urogenital tract and rectal microflora profiles of infertile patients, as well as the microbiota that might have an impact on female infertility and pregnancy outcomes, reflecting the potential impact of microorganisms on female infertility.

The female reproductive tract contains an active microbiome comprising mainly bacteria from the *Lactobacillus* genus, which is associated with a healthy microbiome state (Moreno and Simon, 2019). *Lactobacillus* plays an important role in preventing host illness, including bacterial vaginosis, yeast vaginitis, urinary tract infection, and sexually transmitted diseases (Borges et al., 2014). The capacity of *Lactobacillus* to adhere and compete for adhesion sites in the vaginal epithelium and the capacity to produce antimicrobial compounds (hydrogen peroxide, lactic acid, bacteriocin-like substances), are important in the impairment of colonization by pathogens (Borges et al., 2014). A study found that infertile women show characteristic variations in vaginal microbiome, such as the increased abundance of *Atopobium*, *Aerococcus*, and *Bifidobacterium* and decreased abundance of *Lactobacillus* and *Leuconostoc* (Zhao et al., 2020). In the unexplained infertility patients, the colonization rate of *Lactobacillus* in vaginal samples was lower, and the mean proportion of *Lactobacillus*/total bacterial was significant reduction (Sezer et al., 2022).

In this study, fewer *Lactobacillus* were detected in the vagina and cervix of infertile patients, while the abundance of *Gardnerella* and *Atopobium* increased, which reflected the role of *Lactobacillus* in female reproduction. Since a lower prevalence of *Lactobacillus* and a higher prevalence of anaerobes bacteria including *Gardnerella vaginalis*, *Megasphaera* spp. and *Atopobium vaginae* are the characteristics of bacterial vaginosis, which can lead to many adverse health outcomes, so, it is not surprising that bacterial vaginosis is more common in infertile women and associated with reduced rates of conception (Ravel et al., 2021; Saraf et al., 2021). Therefore, this study suggested that the imbalance of reproductive tract microbiota characterized by *Lactobacillus* reduction may lead to female infertility. Due to the anatomical continuity of the vagina and cervix, there might be a correlation between the microbiota structure of the vagina and cervix. The changing trend of cervical microbiota in infertile patients was consistent with that of vaginas. However, the microbial diversity of the cervix was higher than that of the vagina and higher microbial diversity was detected in the cervix of infertile patients. This was consistent with the research of Wang et al., who found that the cervical microbiota of reproductive-age patients receiving *in vitro* fertilization and embryo transfer (IVF-ET) was more diverse than the vaginal microbiota, and the microbial composition was different between the two sites (Wang et al., 2021). Elevated cervical microbial diversity may affect women’s health.

*Lactobacillus* was detected in high numbers in infertile patients, but in lower abundance than in controls, whereas *Gardnerella* was found to be more abundant in infertile patients. This is similar to the microbial changes in the vagina and cervix. Urinary and reproductive systems are closely associated with each other and infections of one system can easily transmit to another (Safarpoor Dehkordi et al., 2020). Sequencing analysis of the bacterial strains isolated from the vagina and bladder in the same woman showed that *Escherichia coli*, *Streptococcus anginosus*, *L. iners*, and *L. crispatus* were highly similar between them (Thomas-White et al., 2018). That suggested an interlinked female urogenital microbiota that was not only limited to pathogens but was also include health-associated microorganisms (Thomas-White et al., 2018). The study found that BV can be diagnosed by detecting microbes in urine (Datcu et al., 2014). In a study of urgency urinary incontinence microbe, the same change rule of *Lactobacillus* and *Gardnerella* was also found (Pearce et al., 2014). Female infertility might also be affected by microbes from the urinary tract.

The human rectal microbiome was associated with multiple gynecologic conditions. These microbiome likely play a role in the gut-brain axis, which further supports a putative association with the spectrum of symptoms associated with disease (Salliss et al., 2022). This study found that the abundance of rectal microorganisms in infertile patients was significantly lower than that in controls. This may be due to the disruption of the ecological balance of rectal flora. Intestinal microbiome alpha-diversity affects human health, and the occurrence of several diseases is often accompanied by a decrease in diversity (Pickard et al., 2017). A loss in species richness in the GI microbiome is a common finding in several disease states (Heiman and Greenway, 2016). Azpiroz et al. found that infertile patients showed a lower microbial richness and increased Firmicutes/Bacteroidetes ratio at the rectal level (Azpiroz et al., 2021).

Due to the close anatomical distance between the female genitourinary tract and rectum, microbial translocation might exist between different parts. Recent studies indicated a possible relationship between the gut and female tract microbiota, associating specific intestinal bacteria patterns with genital female diseases, such as polycystic ovary syndrome, endometriosis, and bacterial vaginosis (Quaranta et al., 2019). The study found that the intestinal microbiota of fertile and infertile people fluctuated significantly, and the resident intestinal microbiota *Escherichia coli* had a negative impact on female pregnancy and pregnancy outcomes (Cools, 2017; Manzoor et al., 2021). In this study, we found that most microorganisms in the rectum and genitourinary tract of infertile patients were positively correlated with each other through microbial interaction networks. *Lactobacillus* in the rectum, vagina, and urethra might affect each other and promote colonization. *Lactobacillus* was a probiotic in the reproductive tract. Its translocation between the rectum and the urogenital tract may play a role as a barrier for women to resist the invasion of external pathogens. The interaction between rectum and genital tract microbes seems to be certain. It was believed that it is possible to change rectal immune status or microbial composition through oral probiotics, thereby affecting vaginal microbiota (Kim et al., 2019). Therefore, understanding the relationship between rectal and vaginal flora might be another way to treat female reproductive tract diseases.

*Geobacillus thermodenitrificans* had differences in different sites of the urogenital tract and might have predictive effects on female infertility. *Geobacillus* might not be friendly to human health. Study found that *Geobacillus* was enriched in the Extrahepatic cholangiocarcinoma and bladder cancer tissues (Liu et al., 2019; Dudeja et al., 2021). Hawkins et al. found that the presence of *Geobacillus* might affect endometrial cancer disparities and pathogenesis (Hawkins et al., 2022). However, there are few studies on the relationship between *Geobacillus* and female infertility. The detection of *Geobacillus* in the genital tract might be a negative factor for female pregnancy. The mechanism of the influence of *Geobacillus* on female pregnancy needs further study. The abundance of *Campylobacter hominis*, *Sneathia sanguinegens*, and *L. Johnsonii* were found to be significantly different between infertile patients and controls at multiple sampling points (rectum, urethra, and vagina). The relative abundance of *L. Johnsonii* was significantly different at multiple urogenital tract sites between infertile patients and controls, all of which were enriched in the control group. The absence of *L. johnsonii* might be detrimental to a woman’s conception. Studies have shown that *L. johnsonii* were one of the most frequently detected bacterial species in the vaginal milieu of reproductive-age women (Mehta et al., 2020). *L. Johnsonii* could inhibit bacterial vaginosis by inhibiting the expressions of COX-2, iNOS, IL-1β, and TNF-α by regulating NF-κB activation and by killing *G. vaginalis* (Joo et al., 2011).

Pelzer et al. showed that not only follicular fluid is not sterile but bacterial species in follicular fluid can have both positive and negative effects on IVF outcomes (Pelzer et al., 2011, 2013a). The most prevalent microbiota detected in colonized follicular fluids was *Lactobacillus* spp. (*L. crispatus*, *L. gasseri*, *Actinomyces* spp., and *Propionibacterium* spp.; Schoenmakers et al., 2019). Studies have found that *Propionibacterium acnei* and *L. gasseri* (high-dose) in follicular fluid bacteria may result in poor-quality oocytes and/or embryos, leading to poor IVF outcomes (Pelzer et al., 2013b). The presence of *Lactobacillus* on the left and right sides of ovarian follicular fluid was associated with an increased rate of embryo transfer (Pelzer et al., 2013a). In this study, a large number of *L. iners* were detected in follicular fluid, vagina, and cervix of infertile patients. *L. iners* can be detected under normal conditions as well as during vaginal dysbiosis, such as bacterial vaginosis, so the role of this species in reproductive tract health was unclear (Petrova et al., 2017). The study found that *L. iners*-dominated vaginal microbiota appears to be associated with an increased risk of the development of specific pregnancy pathologies (Mls et al., 2019). The presence of *L. iners* in the cervix may be associated with negative ART outcomes (Villani et al., 2022). But there are also studies suggested that the presence of *L. iners* is good for women’s health. Studies have found that the high presence of *L. iners* may prevent the recovery of pathogenic bacteria associated with BV (Srinivasan et al., 2010). The proportion of *L. iners*/*L. brevis* in vaginal swabs of fertile women was significantly higher than that of infertile women (Azpiroz et al., 2021). Endometriosis has been reported to cause infertility, but a study found that a more diverse cervical microbiome may be beneficial for patients to have better clinical outcomes (Chang et al., 2022). However, the opposite conclusion was found in the present study, where the cervix microbiome was higher in the Non-pregnant group than in the pregnant group, although there was no statistical difference. This might be due to the increase in microbial diversity caused by the decrease in *Lactobacillus* abundance.

In this study, *L. acidophilus* was found to be enriched in the follicular fluid of women in Non-pregnant group and was also more abundant in the cervix and vagina than in the pregnant group. Previous studies had suggested that *L. acidophilus* was a probiotic for humans. *L. acidophilus* maintained vaginal health in women by producing more D-lactic acid (Pramanick and Aranha, 2020). However, the presence of *L. acidophilus* in the present study seems to be detrimental to the progress of pregnancy after IVF. Vaginal *L. acidophilus* showed significant probiotic effects on BV and AV development (Pacha-Herrera et al., 2022). However, Kwasniewski et al. found that the cervical community types of Low-grade Squamous Intraepithelial Lesion patients were dominated by *L. acidophilus* and *L. iners* (Kwasniewski et al., 2018). The presence of *L. acidophilus* does not always seem to be beneficial for women, and the mechanism needs to be further investigated.

There are many problems leading to female infertility, and a disorder at any one of the many reproductive steps reduces the chances of pregnancy. A large number of previous studies have linked female infertility with female reproductive tract microflora and body conditions and attempted to explore the mechanism of urogenital tract microflora. The health of the urogenital microbiome is dependent on the health of its individual niche environments and on several intrinsic and extrinsic host factors including pH, hormone levels, daily lifestyle activities, and more (Nakama et al., 2022). In this study, compared with the relatively healthy population, the changes in the urogenital microflora of infertile patients developed towards the trend of the microflora composition that was identified as unfavorable to the human body by previous studies. This change is normal in view of current scientific interpretation, but the mechanism has not been clarified. The reason for this differential microbiome may be related to the patient’s history of illness or trauma, poor lifestyle habits, or even a genetic condition that makes it difficult to conceive.

In this study, the BMI of the infertile patients was lower than that of the healthy controls, which may be related to the fact that the infertile patients carried out a diet conforming to the standards of pregnancy and appropriate exercise to improve their physical fitness during the preparation of pregnancy. Due to the refusal of the healthy control group to collect blood and the unavailability of some private information about the patients, we could not collect more information about the physical conditions of the patients in this study to further explain the reasons for the difference in microflora. In the future study, we will further complete the patient information collection system. Better integration of patient’s health status and urogenital microbiota to further explain the microbial impact on female infertility. Due to the small sample size and the lack of follow-up validation experiments, the results of this study could not determine that there was a causal relationship between the imbalance of the female urogenital tract and rectal microbial community and female infertility. Because the sampling sites are close or continuous in anatomical position, there was a possibility of cross-contamination in the experiment. Future research should develop a sampling method that can deal with this problem.

## Conclusion

In conclusion, this study revealed the characteristics of the urogenital tract and rectal microbial diversity of female infertile patients and the differences between them and controls by 16S rRNA sequencing. Female genitourinary tract and rectum microbes and their interactions might be involved in the pathogenesis of female infertility. Grasping the change rule and potential mechanism of these microorganisms might play a certain role in the intervention of infertility from the perspective of microorganisms.

## Data availability statement

The datasets presented in this study can be found in online repositories. The names of the repository/repositories and accession number(s) can be found at: https://www.biosino.org/node/, OEP003637.

## Ethics statement

The studies involving human participants were reviewed and approved by Medical Ethics Committee of Kunming University of Science and Technology. The patients/participants provided their written informed consent to participate in this study.

## Author contributions

Y-HD, ZF, and N-NZ performed experiments and analyzed data. J-YS: clinical samples collection and DNA extraction. JS: sample preparation and statistical analysis. EY: statistical analysis. Z-MZ: sequencing data analysis. S-YS: clinical samples collection. AX: sample preparation. C-JL: funding acquisition, and writing—review and editing. X-RL: funding acquisition, writing—review and editing, and project administration. All authors contributed to the article and approved the submitted version.

## Funding

This work was supported by the Yunnan Province Innovation Team of Intestinal Microecology-Related Disease Research and Technological Transformation (China; 202005AE160010), Fundamental Application Research Foundation of Yunnan Province (202101AY070001-259), and Yunnan (Kunming) Zhang Wenhong expert workstation (YSZJGZZ-2020051).

## Conflict of interest

S-YS and Z-MZ were employed by LONGEN Technology Group Co., Ltd.

The remaining authors declare that the research was conducted in the absence of any commercial or financial relationships that could be construed as a potential conflict of interest.

## Publisher’s note

All claims expressed in this article are solely those of the authors and do not necessarily represent those of their affiliated organizations, or those of the publisher, the editors and the reviewers. Any product that may be evaluated in this article, or claim that may be made by its manufacturer, is not guaranteed or endorsed by the publisher.
